# Hashimoto's Encephalopathy as a Cause of Persistent Myoclonic Jerks: A Report of a Unique Case

**DOI:** 10.7759/cureus.94980

**Published:** 2025-10-20

**Authors:** Haneen A Salama, Hamza Rastanawie, Ali Deif, Muhammad Umair, Peter G Bernad

**Affiliations:** 1 Faculty of Medicine, University of Benghazi, Benghazi, LBY; 2 Neurology, Aleppo University, College of Medicine, Aleppo, SYR; 3 Faculty of Medicine, Khalifa University, Abu Dhabi, ARE; 4 Faculty of Medicine, Jinnah Sindh Medical University, Karachi, PAK; 5 Neurology, Neurology Services, Inc, Washington, DC, USA

**Keywords:** anti-thyroglobulin antibody, anti-thyro-peroxidase antibody, hashimoto’s encephalopathy, hashimoto’s thyroiditis, myoclonic jerk

## Abstract

Identifying the cause of frequent episodes of myoclonic jerks accompanied by non-specific symptoms in a previously healthy adult can be challenging, especially after ruling out common pathologies such as epilepsy. This case emphasises the importance of considering autoimmune etiologies, particularly Hashimoto's encephalopathy (HE), in the differential diagnosis of encephalopathy.

We present a case of a 45-year-old female patient with a 10-month history of generalised myoclonic jerks, headaches, and word-finding difficulties, all occurring without any alteration in consciousness. Her physical and neurological examinations, brain MRI, 72-hour EEG, and EMG results were unremarkable. However, thyroid peroxidase and anti-thyroglobulin antibody titers were markedly elevated, despite an euthyroid profile. The patient was initiated on oral corticosteroids, resulting in significant clinical improvement.

HE, also known as steroid-responsive encephalopathy associated with autoimmune thyroiditis, is a rare neurological disorder that is linked to Hashimoto's thyroiditis. It typically follows an acute or subacute course and often responds dramatically to corticosteroid therapy, a response that is considered a key diagnostic criterion.

## Introduction

In 1966, Dr. Lord Brain and his colleagues were the first to describe a patient with what is now known as Hashimoto's encephalopathy (HE), reporting on a man who presented with various neurological symptoms [1]. This condition is uncommon, with a prevalence of approximately 2.1 cases per 100,000 individuals, making it a rare neuropsychiatric syndrome more common in women [2]. The diagnosis is associated with serologic evidence of antithyroid antibodies (ATAs) when other causes of encephalopathy are excluded [3]. Currently, HE remains poorly understood, and its pathogenesis is uncertain. Available evidence suggests an autoimmune etiology; however, this has yet to be proven [3]. The occurrence of the disease is not associated with age. Two types of initial clinical presentation may be observed: a vasculitic type with stroke-like episodes and mild cognitive impairment and a diffuse progressive type with predominant dementia [2]. However, some cases cannot be clearly classified into one of these types. For example, Reyes and colleagues previously published the case of a 30-year-old woman diagnosed with HE who predominantly experienced cerebellar and emotional pseudobulbar symptoms [4]. Diagnostic challenges in HE are significant due to its varied presentations, often mimicking other neurological conditions, and the lack of a single definitive diagnostic marker. This case report further expands on the diverse presentations of HE by highlighting a patient whose primary and most challenging symptom was persistent myoclonic jerks, a less commonly emphasized dominant feature, thereby distinguishing it from previously reported cases and more typical presentations of HE.

This case report is intended for submission as an abstract to the AAN annual meeting, scheduled for April 2026 in Chicago, Illinois.

## Case presentation

A 45-year-old, previously healthy, right-handed woman presented to the neurology clinic in January 2025 with headaches, word-finding difficulty, memory problems, and jerking movements of the entire body, which began in August 2024. The patient’s primary concern was the jerks; she described them not as a significant cause of dysfunction, but rather as a significant contributor to persistent fatigue. The patient described the jerks as a slow buildup of energy that would burst and cause a severe sense of fatigue afterward. She also noted that the jerks interfered with her sleep, leaving her awake most of the night. These occurred every day, ranging from every few minutes to every few hours. She denied other significant neurological deficits, such as loss of consciousness, urinary incontinence, tongue biting, weakness, tingling, or numbness. She denied any history of head injury. She had been healthy, though she had contracted COVID-19 four times; the most recent bout was very severe and occurred before the onset of this constellation of symptoms. Her past medical history consists of esophagitis, for which she takes pantoprazole, an appendectomy, and viral myocarditis from COVID-19. The patient’s family history is unremarkable. She is employed, married with children, and does not smoke or drink.

At her most recent visit in May 2025, the patient's vital signs showed a blood pressure of 141/84 mmHg and a pulse of 69. The physical examination was remarkable for persistent whole-body jerks and word-finding difficulty. Muscle power and tone were normal in all extremities. Sensation to pinprick and vibration was intact, and the patient demonstrated 2+ reflexes diffusely. The patient demonstrated normal finger-to-nose, gait, and Romberg testing, in addition to a normal ophthalmologic examination.

The patient underwent a comprehensive assessment starting in February 2025. Initial testing consisted of a brain MRI with and without contrast, EEG, and nerve conduction studies to assess for structural anomalies and epilepsy, all of which yielded normal findings. She was then scheduled for a follow-up appointment to undergo EMG and polysomnogram testing, both of which were inconclusive for any structural or functional abnormalities. Furthermore, the patient underwent extensive blood testing for autoimmune and metabolic disorders, the findings of which can be found in Table 1. The blood work was significant for positive antinuclear antibodies in a speckled pattern (but negative anti-dsDNA), hypercholesterolemia, and elevated thyroglobulin and thyroid peroxidase antibody levels. She was then scheduled for a thyroid ultrasound, which demonstrated mild enlargement of the thyroid gland with no vascularity and two <1 cm nodules in the right lobe.

**Table 1:** Summary of all relevant investigations done in this case

Category	Date	Test	Result	Normal Range
Imaging/Neurophysiology	February 2025	MRI of the brain with and without contrast	Unremarkable	-
		20-minute EEG	No epileptiform discharges	-
		Nerve conduction studies	Unremarkable	-
		EMG	Unremarkable	-
Bloodwork/Autoimmune Markers	March 2025	Blood serum analysis	Unremarkable except for a high cholesterol level (253 mg/dl) and elevated Antinuclear antibody (Speckled): (1:160), negative for anti-dsDNA	<200 mg/dl (for total cholesterol)
Imaging/ Neurophysiology	March 2025	Polysomnogram	Unremarkable	-
		Electronystagmography	Abnormal saccadic movements	-
Thyroid Function	March 2025	Thyroid-stimulating hormone	Normal (3.28 uIU/mL)	0.27-4.2 uIU/mL
		Thyroxine	Normal (8.6 ug/dL)	4.5-12.0 ug/dL
		Triiodothyronine	Normal (111 ng/dL)	80-200 ng/dL
		Thyroglobulin antibody	Elevated (3.9 IU/mL)	0.0-0.9 IU/mL
		Thyroid peroxidase antibody	Elevated (72 IU/mL)	<9 IU/mL
Autoimmune Markers	June 2025	Autoimmune Panel	Normal (Negative MPO, PR3, ANA, ds-DNA)	-
Imaging/Neurophysiology	June 2025	72-hour EEG	No epileptiform discharges.	-
Thyroid Function	June 2025	Thyroglobulin antibody	Elevated (2 IU/mL)	0.0-0.9 IU/mL
		Thyroid peroxidase antibody	Elevated (30 IU/mL)	<9 IU/mL
		Thyroid-stimulating hormone	Normal (1.53 uIU/mL)	0.27-4.2 uIU/mL
		Free thyroxine	Normal (1.08 ng/dL)	0.69-1.48 ng/dL
		Triiodothyronine	Normal (84 ng/dL)	80-200 ng/dL

**Figure 1:**
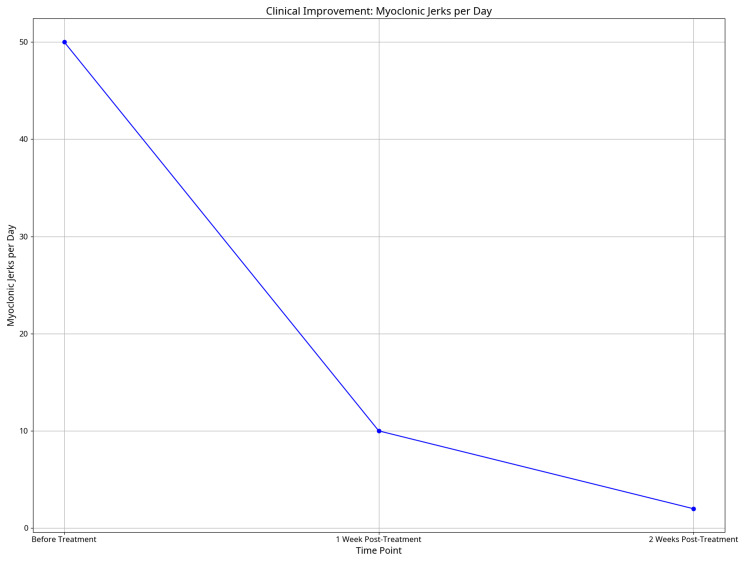
Clinical improvement graph

In March 2025, the patient was prescribed lorazepam and cenobamate, which did not improve her symptoms. In June 2025, she underwent a 72-hour EEG, which yielded normal findings. Considering the patient’s chart and clinical presentation, a provisional diagnosis of HE was made, and she was initially started on an 8mg oral dose of methylprednisolone. This was gradually increased based on clinical response, and after achieving significant improvement, the dose was gradually withdrawn over a three-week period while her symptoms were closely monitored. On a subsequent follow-up, the patient reported significant improvement after one week of treatment; she described a significant decrease in the intensity and frequency of the jerks, from approximately 50 jerks per day before treatment to 10 per day. After two weeks of treatment, she continued to improve, describing a further decrease in the myoclonic jerks to as low as two per day (Figure 1). Blood tests were repeated and yielded normal thyroid function. Thyroid antibody testing showed positive but reduced antithyroglobulin antibodies, and similar findings were noted for anti-thyroid peroxidase antibodies. The autoimmune assay in this round was negative. The patient remains under close long-term monitoring for any signs of symptom relapse.

## Discussion

This study reports a rare presentation of HE that meets the diagnostic criteria for probable HE, including altered mentation, rapidly progressive cognitive impairments, or myoclonus of unknown etiology; absence of hyper- or hypothyroidism symptoms; normal free T4 levels; positive ATA status; absence of other definite autoimmune disorders; and no evidence of paraneoplastic or autoimmune limbic encephalitis antibodies [5].

Typically, there are no significant abnormalities in basic laboratory tests. Inflammatory markers are negative, and patients do not exhibit fever. Thyroid-stimulating hormone (TSH) levels are generally normal, and the degree of thyroid function does not correlate with clinical status. In more than 70% of reported cases, patients were either euthyroid or subclinically hypothyroid, with all having elevated serum antithyroid peroxidase antibodies (anti-TPOAb) and/or anti-thyroglobulin antibodies (anti-TGAb) [6].

In this case report, no laboratory or clinical evidence suggested hypo- or hyperthyroidism; the levels of TSH, T3, and total T4 were within normal limits, measuring 3.28 μIU/mL, 111 ng/dL, and 8.6 μg/dL, respectively. The ATAs were remarkably elevated, with thyroglobulin antibodies four times the upper limit of normal and thyroid peroxidase antibodies twice the upper limit. A cerebrospinal fluid (CSF) examination was not performed due to the patient's frequent and unpredictable myoclonic jerks, which rendered the procedure unsafe at that time. Importantly, CSF testing is not mandatory for the diagnosis of HE, as findings are often nonspecific [3-7]. Normal or mildly elevated white blood cell counts (≤10 cells/mm³, lymphocytic) and protein levels (<100 mg/dL), along with ATAs, can be present in some patients [7]. Therefore, the absence of CSF analysis does not undermine diagnostic confidence in this case.

Normal brain MRI findings are common among patients diagnosed with HE, with 51% of diagnosed cases showing no abnormalities [3-7]. Although MRI is not a crucial diagnostic criterion, it is necessary to exclude other conditions that may mimic HE, such as neoplastic, vascular, or inflammatory processes. In this case report, a brain MRI with and without contrast was performed in February 2025 as part of the initial patient assessment and revealed completely normal and unremarkable results. However, the neurological symptoms began five months before the MRI was conducted, presenting a limitation, as abnormal MRI findings may be challenging to detect without using FLAIR techniques after such a duration.

Two types of initial clinical presentations may be observed in HE: a vasculitic type characterized by stroke-like episodes and cognitive impairment and a diffuse progressive type with predominant dementia [2-7]. In this case, the primary presenting symptom and the patient's greatest concern were recurrent myoclonic jerks. There were no stroke-like symptoms, ataxia, weakness, or permanent cognitive decline. Cerebellar signs, including gait ataxia, dysmetria, and nystagmus, have also been described in HE patients, sometimes dominating the clinical picture [8]. Initially, the patient experienced headaches, poor concentration, and memory issues, accompanied by word-finding difficulties. In this context, limbic encephalitis does not explain the entire clinical picture. Despite early memory issues, the patient showed spontaneous improvement, which was not associated with psychosis, hallucinations, or mood changes; however, she reported anxiety and stress due to her debilitating myoclonic jerks throughout the day.

As HE is diagnosed by exclusion, it was vital to rule out other autoimmune etiologies. Consequently, a blood autoimmune panel was requested and found to be within normal limits (negative MPO, PR3, ANA, and ds-DNA findings). Although the detection of anti-TPO and anti-TG antibodies is crucial for diagnosing HE, these markers are nonspecific and can be present in approximately 10% of healthy young individuals and 15% of those over 60 years of age. The role of thyroid autoantibodies in central nervous system (CNS) tissue damage remains unclear; therefore, they may only serve as diagnostic markers. Recent case reviews suggest that these antibodies may cross-react with cerebellar or other CNS antigens, potentially contributing to neurological dysfunction [9]. Conversely, they may play a role in determining thyroid pathology. Since there is no direct pathophysiological link between ATAs, Hashimoto's thyroiditis, and the cerebral syndrome, the nomenclature of HE can be misleading. The response to steroids has led to the renaming of the syndrome to steroid-responsive encephalopathy associated with autoimmune thyroiditis (SREAT), although some cases do not respond to steroids [3]. In steroid-resistant cases or when steroids are contraindicated, alternative immunotherapies may be effective. Intravenous immunoglobulin (IVIG), administered at 2 g/kg over 2-5 days, has been associated with rapid neurological improvement in several recent cases, including a systematic review of 14 HE cases demonstrating favorable outcomes after IVIG therapy [10]. Plasma exchange, azathioprine, and emerging biologics, such as rituximab, have also shown success; however, controlled trials are limited, and protocols remain to be standardized [11].

With recent advancements and new insights in immunology and autoimmune neurological disorders, this entity may be amalgamated with other disorders in the future. However, for the time being, any clinical syndrome characterized by extremely high anti-TPO antibodies, distinctive clinical features, and clinical improvement following steroid administration is regarded as HE. In this relevant case, the titer of anti-TPO was decreased to normal levels (30 IU/mL) one week after initiating corticosteroids. This finding, along with the significant clinical response to corticosteroids, supports our diagnosis of HE.

## Conclusions

This case highlights the diagnostic complexity of HE, particularly in euthyroid patients with normal thyroid hormone levels and unremarkable neuroimaging. Notably, this presentation was dominated by persistent myoclonic jerks, a feature that, while recognized in HE, was the primary and most challenging symptom in this case, underscoring its potential as a prominent manifestation. Despite normal TSH, T3, and T4 values, the diagnosis was strongly supported by markedly elevated ATAs, consistent with an autoimmune etiology. The absence of a CSF analysis due to persistent myoclonic jerks did not hinder the diagnosis, as CSF findings in HE are often nonspecific and not required for confirmation. Similarly, the normal brain MRI, performed several months after symptom onset, did not exclude HE, as more than half of patients may show no abnormalities on imaging.

Importantly, the patient demonstrated significant clinical improvement following corticosteroid therapy, further supporting the diagnosis of SREAT. This case underscores the importance of considering HE in patients with unexplained neuropsychiatric symptoms, especially when persistent myoclonus is a prominent feature, even in the absence of thyroid dysfunction or definitive imaging findings. Long-term management will involve careful tapering of corticosteroids and vigilant monitoring for potential relapses, given the chronic and relapsing nature of HE.
